# Relationship between right heart catheterization and exercise testing in heart failure patients with and without pulmonary hypertension – cross-sectional study

**DOI:** 10.3389/fcvm.2026.1812393

**Published:** 2026-08-18

**Authors:** Filip Sawczak, Agata Kukfisz, Magdalena Dudek, Ewa Straburzyńska-Migaj, Marta Kałużna-Oleksy

**Affiliations:** 1 College of Medical Sciences, SGMK Copernicus University, Olsztyn, Poland; 2 1st Department of Cardiology, Poznan University of Medical Sciences, Poznan, Poland; 3 3rd Department of Cardiology, Silesian Center for Heart Diseases, Medical University of Silesia, Zabrze, Poland

**Keywords:** exercise capacity, heart failure with reduced ejection fraction, left heart disease, pulmonary hypertension, systolic dysfunction

## Abstract

**Introduction:**

Left-sided heart disease is the leading cause of pulmonary hypertension (PH). Right heart catheterization (RHC) is the gold standard for diagnosing PH, although it is invasive. Noninvasive cardiopulmonary exercise testing (CPET) could potentially help select heart failure (HF) patients who require RHC.

**Objectives:**

The study aimed to assess whether CPET could predict PH in patients with HF.

**Patients and methods:**

In this prospective cohort study, we analyzed data from 428 HF patients with a median age of 55 years and reduced left ventricular ejection fraction (≤40%) who underwent both RHC and CPET. Patients with acute clinical instability or precapillary PH were excluded.

**Results:**

The median LVEF was 20%, and 66.8% of patients were in NYHA class III or IV. PH was diagnosed by RHC in 76.9% of patients. Breathing reserve was lower and peak respiratory exchange ratio was higher in patients with PH. Patients with pulmonary vascular resistance (PVR) > 3 WU and mean pulmonary artery pressure >40 mmHg had lower indexed peak oxygen uptake and higher VE/VCO2. Indexed peak oxygen uptake improved the prediction of PH with PVR > 3 WU when added to the model composed of clinical and laboratory parameters.

**Conclusions:**

No differences were observed in indexed peak oxygen uptake or VE/VCO₂ slope between patients with and without PH. However, CPET results allowed more accurate prediction of more severe PH phenotypes. Patients with PH had lower ejection fraction and worse NYHA functional class and were more likely to be male and to have chronic kidney disease and diabetes. Overall, CPET parameters correlated with RHC findings and may help identify HF patients with PH, especially with high pulmonary vascular resistance. However, CPET cannot replace invasive hemodynamic assessment by RHC.

## Graphical Abstract

**Figure:**
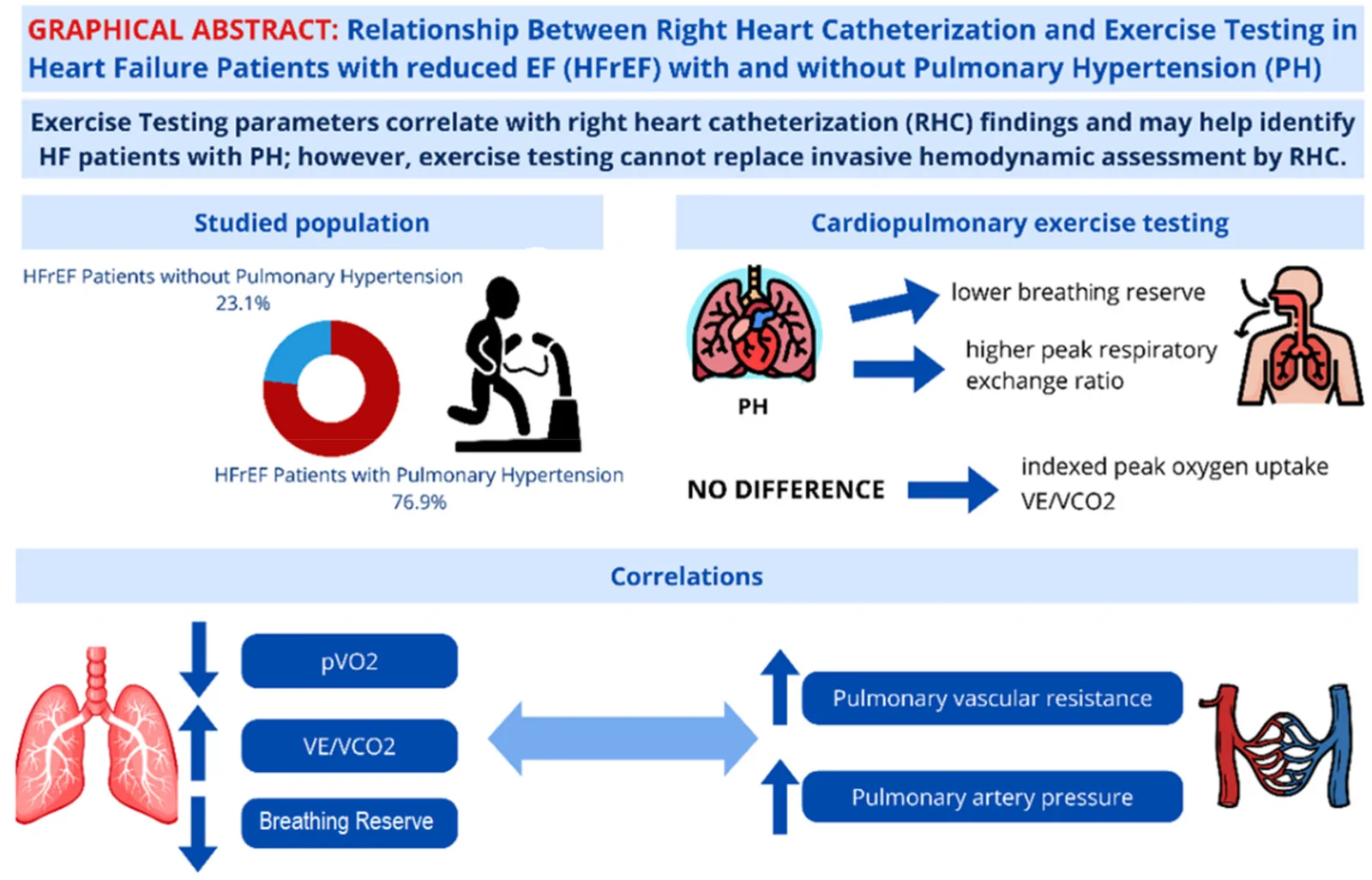

## 1 Introduction

Heart failure (HF) and pulmonary hypertension (PH) are both major global health problems, with a prevalence of 1%–2% (1) and 1% (2) of the world's population, respectively. PH is more prevalent in individuals older than 65 years (2, 3), largely due to its major underlying causes, including left heart disease and chronic lung disease (2, 3). Left heart disease represents the most common cause of PH, accounting for nearly 50% of cases (3). It is a heterogeneous condition that includes systolic or diastolic left ventricular dysfunction as well as valvular heart disease. Up to 60% of patients with severe systolic dysfunction and up to 70% of patients with isolated diastolic dysfunction develop PH (4). Importantly, PH associated with left heart disease is linked to a worse prognosis (4, 5). Right heart catheterization (RHC) is the gold standard for the diagnosis and classification of PH (3) and is an essential component of the evaluation for heart transplantation (6). Identifying PH is critical, as severe and irreversible PH represents a contraindication to heart transplantation; in such patients with advanced HF, left ventricular assist device therapy should be considered instead (6).

Although elevated left-sided filling pressures can be estimated by Doppler echocardiography, invasive measurement of pulmonary artery pressure (PAP) and pulmonary arterial wedge pressure (PAWP) is required to confirm the diagnosis of PH due to left heart disease (3). RHC, as an invasive diagnostic tool, is not widely available and is often unsuitable for routine follow-up because of the risk of rare but potentially serious complications, including bleeding and pneumothorax (7). Therefore, there is a need for noninvasive tools to identify patients whose diagnostic and therapeutic management would benefit from RHC. Such tools should be practical and allow for repeated assessments over time. Cardiopulmonary exercise testing (CPET) is one of the tools used in the evaluation of patients with HF that may facilitate such selection (8). It is a noninvasive test that is routinely performed in the assessment of HF patients and in their evaluation for heart transplantation, providing objective information on exercise capacity (8).

The aim of this study was to assess whether CPET could predict PH and to compare CPET findings between HF patients with and without PH. In addition, we evaluated the associations between RHC parameters and CPET findings in this population.

## 2 Methods

### 2.1 Participants and data collection

The study is based on a prospective registry of patients with HFrEF undergoing RHC. Most patients were admitted to the Cardiology Clinic for elective evaluation for heart transplantation between January 2013 and September 2020. The inclusion criteria were HFrEF, defined as LVEF ≤40%, and RHC performed during hospitalization. The exclusion criteria were absence of CPET during hospitalization, acute clinical instability, and a diagnosis of precapillary PH. The analyses included medical history, demographic data, selected laboratory test results, including N-terminal pro–B-type natriuretic peptide and B-type natriuretic peptide, echocardiographic parameters including LVEF, as well as CPET and RHC findings.

PH was diagnosed in patients with mean pulmonary artery pressure (PAP) > 20 mmHg, according to the 2022 European Society of Cardiology/European Respiratory Society guidelines (3). Isolated postcapillary PH was diagnosed if pulmonary arterial wedge pressure was >15 mmHg and pulmonary vascular resistance (PVR) was ≤2.0 Wood units, whereas combined pre- and postcapillary PH was defined by a wedge pressure >15 mmHg and PVR >2.0 Wood units (3).

Patients underwent maximal, symptom-limited treadmill CPET. Heart rate was monitored continuously by electrocardiography at rest and during exercise. Blood pressure was measured with a sphygmomanometer every 3 min during the test, at peak exercise, and at least twice during recovery. Respiratory gas analysis was performed using a metabolic cart (Vmax ENCORE), which was calibrated with known concentrations of standard gases before each test. Peak oxygen uptake and peak respiratory exchange ratio were defined as the highest 30-second average values obtained during exercise. The VE/VCO₂ slope was calculated by linear regression using exercise data obtained up to the anaerobic threshold (AT).

All patients underwent RHC performed by a physician who was unaware of the results of noninvasive diagnostic tests. RHC was performed using a Swan–Ganz catheter. The catheter was inserted through a vascular access sheath under fluoroscopic guidance via the superior or inferior vena cava into the right atrium to measure right atrial pressure. It was then advanced through the tricuspid valve into the right ventricle to measure systolic, diastolic, and end-diastolic pressures. Next, the catheter was advanced through the pulmonary valve into the pulmonary artery to measure systolic, diastolic, and mean PAP. The balloon was then wedged in a pulmonary artery branch to measure pulmonary arterial wedge pressure, which serves as an approximation of left atrial pressure. Cardiac output was measured using the thermodilution method by assessing changes in blood temperature in the pulmonary artery after injection of 10 mL of 0.9% NaCl at approximately 0–5 °C into the right atrium. The average of three comparable measurements was used to calculate cardiac output, which was subsequently indexed to body surface area to obtain the cardiac index.

### 2.2 Ethical consideration

The research protocol was reviewed and approved by the Bioethics Committee of the Medical University in Poznań (approval code 586/24) and was conducted in full compliance with the principles outlined in the Declaration of Helsinki. All participants provided written informed consent prior to enrollment. To protect patient confidentiality, all personal data were anonymized during data handling and analysis.

### 2.3 Statistical methods

Statistical analysis was performed using STATISTICA version 13.3 (TIBCO Software Inc., Palo Alto, CA, USA) and R software (version 4.5.2; R Core Team) and RStudio (Posit, Boston, MA, USA).

The distribution of continuous variables was assessed using the Kolmogorov–Smirnov test. Patients were divided according to the presence of PH, and the two groups were compared. Moreover, patients were divided into groups with PVR >3 WU versus PVR ≤3 WU, with mean PAP ≥25 mmHg versus mean PAP <25 mmHg and these groups were compared (results presented in supplementary data). Student's t test was used for continuous variables with a normal distribution and homogeneity of variance, whereas the Mann–Whitney U test was used for non-normally distributed continuous variables. Categorical variables were compared using chi-square or the two-sided Fisher's exact test as appropriate. Additionally, patients were stratified by PVR into four groups: no PH, PH with PVR <2 WU, (3) PH with PVR 2–3, (4) PH with PVR >3 WU and then compared. Continuous variables were compared between four groups with ANOVA or Kruskal–Wallis test as appropriate. Tukey's or Dunn tests as appropriate were used for *post-hoc* comparisons for selected CPET results. Categorical variables were compared between the four groups using the Pearson chi-square test. Similarly, population was stratified with mean PAP into four groups: (1) no PH, (2) mean PAP >20 mmHg and ≤30 mmHg, (3) mean PAP >30 mmHg and ≤40 mmHg, (4) mean PAP >40 mmHg. Spearman correlation analysis was used to assess associations between CPET results and RHC-derived variables. Receiver operating characteristic curves were analyzed to evaluate the ability of CPET parameters to predict the presence of PH. Furthermore, multivariable models were constructed for PH and PVR >3 WU as dependent variables. First, we calculated models based on the clinical and laboratory parameters with best subset selection method based on Akaike criterion and then augmented them with indexed VO2 max, VE/VCO2 and breathing reserve (one at once) to assess if they can improve prediction. BNP concentrations were not available in 60/429 (14%) cases. Therefore, for multivariable analysis, its values were estimated, in these cases, using the formula developed and tested by Ishihara et al. (9), which was previously reported to have an R^2^ of 0.900.

Continuous variables are presented as mean with standard deviation for normally distributed data and as median with lower and upper quartiles for non-normally distributed data. Categorical variables are presented as numbers and percentages. A *p* value <0.05 was considered statistically significant.

## 3 Results

**Figure 1:**
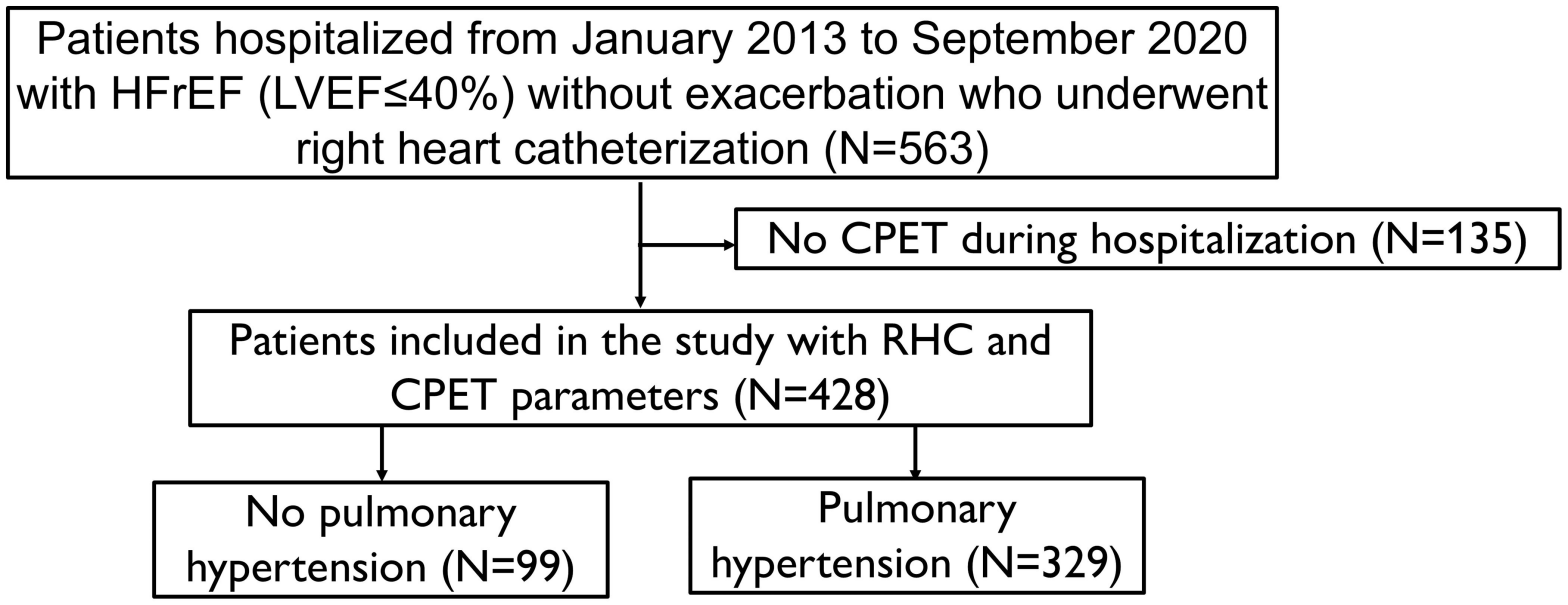
Flowchart demonstrating the enrolment process. CPET - cardiopulmonary exercise testing (CPET), HFrEF - heart failure with reduced ejection fraction, RHC - right heart catheterization.

A total of 428 patients who underwent both RHC and CPET were included in the study, including 329 patients with postcapillary PH and 99 patients without PH (Figure 1; Table 1). The median age was 55 years (range, 24–78 years). Women represented 13% of the study population and were more prevalent in the non-PH group. The median LVEF was 20% (range, 8%–40%) and was lower in patients with PH compared with those without PH. Two-thirds of the population were in NYHA class III or IV, with patients with PH exhibiting a worse NYHA functional class. Ischemic heart disease was the etiology of HF in approximately half of the patients. Chronic kidney dysfunction, systemic arterial hypertension, and atrial fibrillation were the most common comorbidities, each affecting approximately one-third of the population. Chronic kidney dysfunction and diabetes mellitus were more prevalent in the PH group. There were no differences in the prevalence of chronic obstructive pulmonary disease. Patients with PH had larger diameters of both ventricles and right atrium than patients without PH. Regarding complete blood count findings, PH patients had a higher platelet count. Most patients were treated with loop diuretics, beta blockers, mineralocorticoid receptor antagonists, angiotensin-converting enzyme inhibitors, angiotensin receptor blockers, or angiotensin receptor–neprilysin inhibitors. Angiotensin receptor–neprilysin inhibitors were more frequently prescribed in patients without PH. Use of SGLT2 inhibitors was rare, as the study period (2013–2020) preceded guideline recommendations for their use in HF (1).

**Table 1:** General characteristics of the study group. Comparison of patients with and without pulmonary hypertension.

Variable	All patients (*N* = 428)	Patients with pulmonary hypertension (*N* = 329)	Patients without pulmonary hypertension (*N* = 99)	*p* value
Age (years)	55 (45–60)	55 (46–60)	54 (44–60)	0.60
Female sex	57 (13.3%)	37 (11.2%)	20 (20.2%)	0.021
Body mass index (kg/m2)	26.96 (4.02)	27.12 (3.95)	26.43 (4.26)	0.13
Ischemic etiology	212 (49.5%)	168 (51.1%)	44 (44.4%)	0.248
Heart rate during RHC	70 (63–76)	70 (65–80)	66 (60–75)	<0.001
(beats/min)				
NYHA class				
I	4 (0.9%)	3 (0.9%)	1 (1.0%)	0.011
II	138 (32.2%)	93 (28.3%)	45 (45.4%)	
III	242 (56.5%)	195 (59.3%)	47 (47.5%)	
IV	44 (10.3%)	38 (11.6%)	6 (6.1%)	
III or IV	286 (66.8%)	233 (70.8%)	53 (53.5%)	0.001
Comorbidities				
Diabetes mellitus	111 (25.9%)	93 (28.3%)	18 (18.2%)	0.045
Chronic kidney dysfunction (GFR<60 mL/min/1.73m2)	147 (34.4%)	122 (37.1%)	25 (25.2%)	0.030
COPD	42 (9.8%)	30 (9.1%)	12 (12.1%)	0.379
AF at hospitalization	84 (19.6%)	68 (20.7%)	16 (16.2%)	0.322
History of paroxysmal AF	62 (14.5%)	47 (14.3%)	15 (15.2%)	0.830
Systemic arterial hypertension	148 (34.6%)	116 (35.3%)	32 (32.3%)	0.577
Implanted ICD or CRTD	262 (61.2%)	199 (60.5%)	63 (63.6%)	0.362
Medications				
Loop diuretics (%)	413 (96.5%)	318 (96.7%)	95 (96.0%)	0.757
β-blockers (%)	409 (95.6%)	312 (94.8%)	97 (98.0%)	0.267
ACEI or ARB (%)	353 (82.5%)	270 (82.1%)	83 (83.8%)	0.684
ARNI (%)	37 (8.6%)	23 (7.0%)	14 (14.1%)	0.026
MRA (%)	377 (88.1%)	287 (87.2%)	90 (90.9%)	0.322
Laboratory parameters				
BNP (pg/mL)	469 (223–891)	570 (304–1,004)	150 (83–368)	<0.001
NT-proBNP (pg/mL)	1,999 (989–3,992)	2,282 (1,355–4,526)	921 (412–2,100)	<0.001
GFR MDRD (mL/min/1.73m2)	70.4 (22.2)	68.5 (21.0)	76.1 (25.4)	0.001
Uric acid [µmol/L]	454.3 (147.2)	467.8 (150.2)	409.7 (127.5)	0.066
Hemoglobin (mmol/L)	8.98 (0.89)	8.96 (0.89)	9.05 (0.90)	0.41
RBC [10^12^/L]	4.82 (4.42–5.08)	4.78 (4.43–5.09)	4.83 (4.41–5.07)	0.788
WBC [10^9^/L]	7.62 (1.91)	7.66 (1.87)	7.49 (2.03)	0.437
PLT [10^9^/L]	200 (164–228)	195 (159–226)	218 (178–242)	0.004
Echocardiography parameters				
LVEF (%)	20 (15–25)	20 (15–25)	25 (20–27)	<0.001
LVEDD (mm)	72.7 (11.4)	73.5 (11.0)	69.8 (12.3)	0.004
RVD (mm)	35.8 (7.3)	36.7 (7.2)	32.7 (7.0)	<0.001
LAD (mm)	53 (48–58)	54 (49–58)	48 (43–53)	<0.001

Categorical variables are presented as number of patients (% of each group), continuous variables with normal distribution are presented as mean ± standard deviation, while other variables are depicted as median (lower quartile - upper quartile). ACEI, angiotensin-converting-enzyme inhibitors; AF, atrial fibrillation; ARB, angiotensin receptor blockers; ARNI, angiotensin receptor-neprilysin inhibitors; BNP, B-type natriuretic peptide; COPD, chronic obstructive pulmonary disease; CRTD, implantable cardiac resynchronization therapy defibrillator; eGFR, glomerular filtration rate; ICD, implantable cardioverter-defibrillator; LAD, left diameter; LVEDD, left ventricular end-diastolic diameter; LVEF, left ventricular ejection fraction; MDRD, Modification of the Diet in Renal Disease; MRA, mineralocorticoid receptor antagonist; NYHA, New York Heart Association classification; NT-proBNP, N-terminal pro-B-type natriuretic peptide; PLT, platelet count; RBC, red blood cell count; RVD, right ventricular diameter; WBC, white blood cell count.

With respect to CPET findings, breathing reserve was lower and peak respiratory exchange ratio was higher in patients with PH compared with those without PH (Table 2). No differences were observed in other CPET parameters, including indexed peak oxygen uptake or VE/VCO₂ slope. However, when alternative hemodynamic thresholds were applied, functional capacity differed between selected groups. Patients with mean PAP ≥25 mmHg (*N* = 292) in comparison with those with mean PAP <25 mmHg had lower indexed peak oxygen uptake and higher VE/VCO2 (Supplementary Table 1). Similar results were observed for subjects with PVR >3 WU (*N* = 122), who had lower functional capacity than patients with PVR ≤3 WU (Supplementary Table 2).   The main reasons for terminating exercise were cardiogenic symptoms, primarily dyspnea and fatigue, while only a few patients discontinued the test for non-cardiac reasons.

**Table 2:** Comparison of right heart catheterization and cardiopulmonary exercise testing results between patients with and without pulmonary hypertension.

Variable	All patients (*N* = 428)	Patients with pulmonary hypertension (*N* = 329)	Patients without pulmonary hypertension (*N* = 99)	*p* value
Right heart catheterization parameters				
Mean pulmonary artery pressure (mmHg)	31.8 (13.1)	36.6 (11.0)	15.9 (3.3)	<0.001
Right atrial mean pressure (mmHg)	7.5 (4–12)	9 (5–13)	3 (2–6)	<0.001
Systemic arterial systolic pressure (mmHg)	114 (103.5–125)	113 (104–125)	115 (103–126.5)	0.76
Systemic arterial mean pressure (mmHg)	91 (84–97)	91 (84–98)	90 (83–97)	0.43
Systolic right ventricle pressure (mmHg)	43 (30–56)	48 (38–60)	25.5 (21–28)	<0.001
Diastolic right ventricle pressure (mmHg)	6 (2–10)	7 (3–10)	1 (1–4)	<0.001
End-diastolic right ventricle pressure (mmHg)	13 (8–20)	15.5 (10–22)	8 (4–11)	<0.001
Cardiac output (L/min)	4.87 (4.10–5.53)	4.78 (4.06–5.50)	5.05 (4.33–5.78)	0.068
Cardiac index (L/min/m2)	2.48 (2.10–2.80)	2.45 (2.10–2.77)	2.60 (2.20–3.0)	0.0069
PAWP (mmHg)	21 (14–26)	23 (19–28)	9 (7–12)	<0.001
Pulmonary vascular resistance (Wood units)	2.0 (1.43–3.26)	2.41 (1.63–3.73)	1.39 (1.04–1.84)	<0.001
Pulmonary vascular resistance (dyn)	160 (115–261)	193 (129–298)	110 (83–147)	<0.001
Transpulmonary pressure gradient (mmHg)	10 (7–14)	11 (8–17)	7 (5–8)	<0.001
Diastolic pulmonary gradient (mmHg)	2.0 [(−1.0)−4.0]	2.0 [(−1.0)−5.0]	1.0 (0–3.0)	0.0068
Cardiopulmonary exercise testing parameters				
Peak oxygen uptake (mL/min)	1,180 (917.5–1,484)	1,183 (934–1,445)	1,179 (890–1,524)	0.87
Indexed peak oxygen uptake (mL/kg/min)	15.00 (4.58)	14.82 (4.45)	15.58 (4.99)	0.15
Tidal volume (mL)	1,566.8 (448.1)	1,577.1 (424.0)	1,548.1 (501.7)	0.58
VE/VCO2 slope	37.2 (29.5–42.5)	37 (30.7–42.5)	34.1 (28.1–42.5)	0.13
Oxygen uptake at anaerobic threshold (mL/min)	847.8 (305.2)	839.6 (305.0)	881.0 (306.1)	0.42
Breathing reserve (%)	40.5 (17.1)	39.5 (16.9)	44.6 (17.4)	0.015
Peak respiratory exchange ratio	1.03 (0.98–1.09)	1.04 (0.99–1.09)	1.02 (0.95–1.07)	0.02

Continuous variables with normal distribution are presented as mean (standard deviation), while other variables are depicted as median (lower quartile - upper quartile).

PAWP, pulmonary arterial wedge pressure; VE/VCO2, minute ventilation to carbon dioxide output.

Studied population was stratified by PVR values to four groups (Table 3.). The indexed peak oxygen uptake was significantly lower in patients with PVR >3 WU than in the groups without PH, with PH and PVR < 2 WU and with PVR 2–3 WU. Subjects in the PVR >3 WU group had a significantly higher VE/VCO₂ slope than those in any other subgroup (Table 3). Similarly, after stratification by mean PAP values patients with mean PAP >40 mmHg had significantly lower peak oxygen uptake compared with those without PH or with mean PAP 21–30 mmHg (Supplementary Table 3). No significant differences were reported between VE/VCO2 between groups stratified by mean PAP (Supplementary Table 3).

**Table 3:** Characteristics of the study group stratified by pulmonary vascular resistance. Continuous variables were compared with ANOVA or Kruskal–Wallis test according to data distribution. Detailed *post-hoc* Tukey or Dunn tests results for indexed peak oxygen uptake, VE/VCO2 slope, breathing reserve are presented below the table.

Variable	Patients without PH (mean PAP ≤20 mmHg (*N* = 99) (A)	Patients with PH and PVR<2 WU (*N* = 130) (B)	Patients with PH and PVR 2–3 WU (*N* = 79) (C)	Patients with PH and PVR >3 WU (*N* = 120) (D)	*p* value
Age (years)	54 (44–60)	53 (43–59)	55 (46–60)	56.5 (49–61)	0.119
Female sex	20 (20.2%)	11 (8.5%)	7 (8.9%)	19 (15.3%)	0.033
Body mass index (kg/m2)	26.43 (4.26)	27.83 (4.24)	27.17 (3.51)	27.44 (3.92)	0.410
Ischemic etiology	44 (44.4%)	65 (50%)	32 (40.5%)	71 (59.2%)	0.045
LVEF (%)	25 (20–27)	20 (15–25)	20 (15–25)	20 (15–25)	0.016
NYHA III/IV	53 (53.5%)	85 (65.4%)	56 (70.9%)	92 (76.7%)	0.006
BNP (pg/mL)	150 (83–368)	461 (265–886)	487 (301–1,135)	679 (418–1,134)	<0.001
NT-proBNP (pg/mL)	921 (412–2,100)	1,998 (1,044–4,767)	2,371 (1,798–4,526)	2,419 (1,510–4,334)	<0.001
GFR MDRD (mL/min/1.73m2)	76.1 (25.4)	69.8 (22.9)	68.0 (20.7)	62.1 (17.9)	<0.001
Hemoglobin (mmol/L)	9.05 (0.90)	8.96 (0.87)	9.03 (0.88)	8.92 (0.94)	0.706
Peak oxygen uptake (mL/min)	1,179 (890–1,524)	1,294 (1,059–1,609)	1,222 (966–1,513)	1,105 (896–1,350)	<0.001
Indexed peak oxygen uptake (mL/kg/min)	15.58 (4.99)	16.09 (5.02)	15.09 (3.90)	13.26 (3.61)	<0.001^a^
Tidal volume (mL)	1,548.1 (501.7)	1,655 (419)	1,615 (442)	1,470 (398)	0.008
VE/VCO2 slope	34.1 (28.1–42.5)	35.2 (28.2–39.9)	34.8 (30.3–41.3)	39.2 (33.3–46.2)	0.005^b^
Oxygen uptake at anaerobic threshold (mL/min)	881 (306.1)	921 (318)	841 (276)	739 (285)	0.005
Breathing reserve (%)	44.6 (17.4)	41.4 (14.5)	39.0 (17.7)	37.5 (18.8)	0.028^a^
Peak respiratory exchange ratio	1.02 (0.95–1.07)	1.02 (0.97–1.09)	1.05 (0.99–1.09)	1.04 (0.99–1.09)	0.086

Categorical variables are presented as number of patients (% of each group), continuous variables with normal distribution are presented as mean (standard deviation), while other variables are depicted as median (lower quartile - upper quartile).

a Tukey *post-hoc* tests results: - Indexed peak oxygen uptake (mL/kg/min): A vs. B *p* = 0.828, A vs. C *p* = 0.885, B vs. C *p* = 0.395, A vs. D *p*=<0.001; B vs. D *p*=<0.001; C vs. D *p* = 0.024, - Breathing reserve (%): A vs. B *p* = 0.625, A vs. C *p* = 0.220, B vs. C *p* = 0.780, A vs. D *p* = 0.043; B vs. D *p* = 0.344; C vs. D *p* = 0.950.

b Dunn *post-hoc* tests results: VE/VCO2 slope: A vs. B *p* = 1.000, A vs. C *p* = 1.000, B vs. C *p* = 1.000, A vs. D *p* = 0.007; B vs. D *p* = 0.001; C vs. D *p* = 0.031.

BNP, B-type natriuretic peptide; eGFR, glomerular filtration rate; LVEF, left ventricular ejection fraction; MDRD, Modification of the Diet in Renal Disease; NYHA, New York Heart Association classification; NT-proBNP, N-terminal pro-B-type natriuretic peptide; PVR, pulmonary vascular resistance; WU, Wood unit; VE/VCO2, ventilatory equivalent for carbon dioxide.

**Figure 2:**
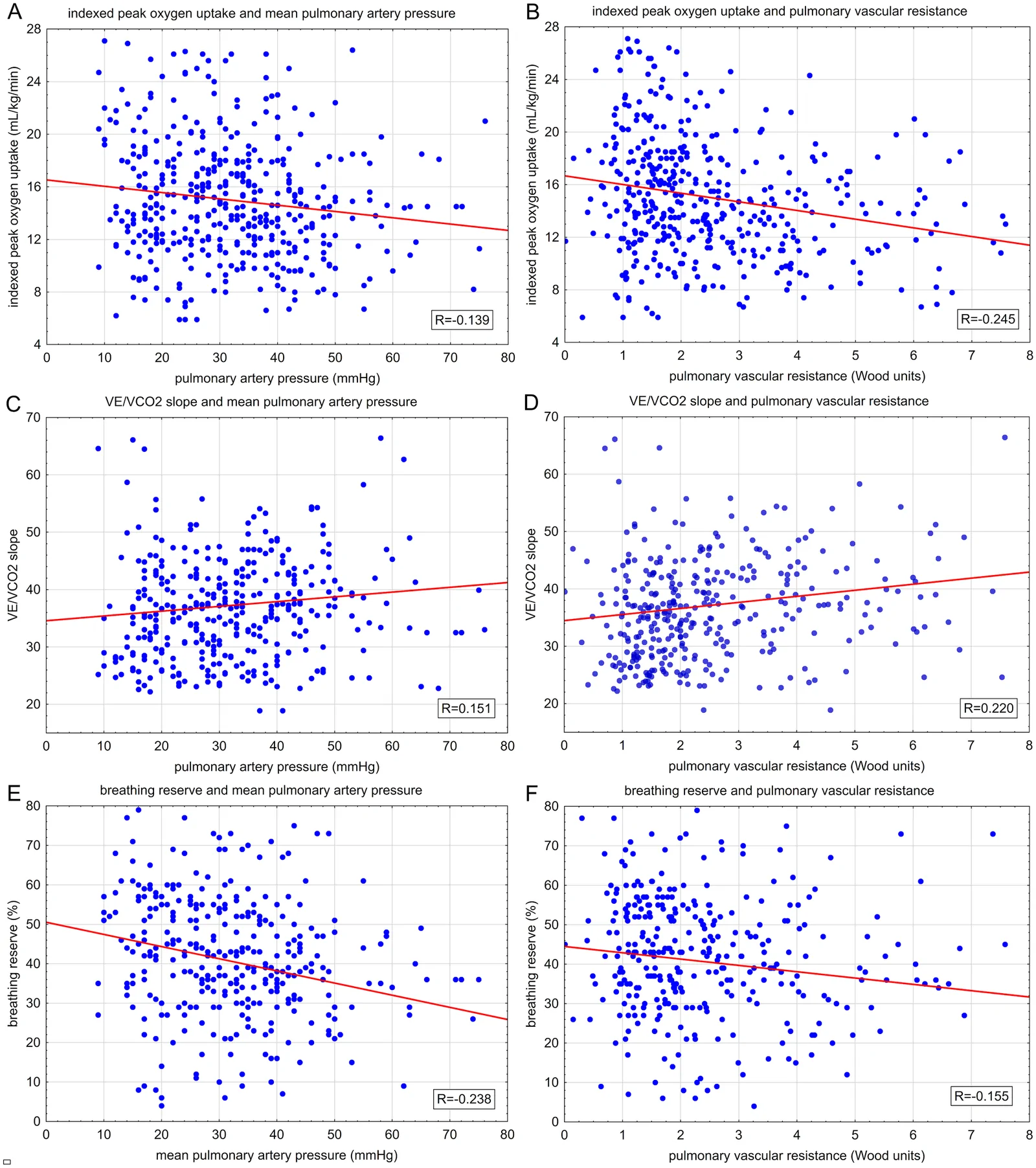
Correlations between mean pulmonary pressure **(A,B,C)**, pulmonary vascular resistance **(D,E,F)** and peak indexed oxygen uptake **(A,B)**, VE/VCO2 **(C,D)** and breathing reserve **(E,F)**. peak VO2 - peak oxygen uptake, VE/VCO2 - minute ventilation to carbon dioxide output.

The correlation analysis between right heart catheterization and cardiopulmonary exercise testing (CPET) parameters exhibited significant interconnections (Table 4; Figure 2). The strongest association between CPET and RHC parameters was observed between nonindexed peak oxygen uptake (mL/min) and cardiac output (L/min) (R = 0.331; *p* < 0.001). For further investigation, please refer to the detailed results (Table 4; Figure 2).

**Table 4:** Correlations between right heart catheterization and cardiopulmonary exercise testing (CPET) parameters.

Variable	peak oxygen uptake (mL/min)	Indexed peak oxygen uptake (mL/kg/min)	Tidal volume (mL)	VE/VCO2	Anaerobic threshold (L/min)	Breathing reserve (%)	Peak RER
Pulmonary hypertension	0.008	−0.057	0.028	0.080	−0.069	−0.133*	0.108*
Mean pulmonary artery pressure (mmHg)	−0.046	−0.139*	0.045	0.151*	−0.082	−0.238**	0.117*
Right atrial mean pressure (mmHg)	−0.056	−0.102*	−0.02	0.102	−0.003	−0.177*	0.114*
Aortic systolic pressure, (mmHg)	0.134*	0.018	0.025	−0.115*	0.088	−0.009	−0.083
Aortic mean pressure (mmHg)	0.121*	0.006	0.058	−0.068	0.084	−0.056	−0.074
Systolic right ventricle pressure (mmHg)	−0.082	−0.194**	−0.022	0.124*	−0.090	−0.201**	0.105*
Diastolic right ventricle pressure (mmHg)	−0.113*	−0.155*	−0.142*	0.075	−0.057	−0.172*	0.055
End-diastolic right ventricle pressure (mmHg)	−0.101*	−0.124*	−0.079	0.111*	−0.116*	−0.143*	0.064
Cardiac output (l/min)	0.332**	0.168**	0.224**	−0.239**	0.275**	0.106	−0.142*
Cardiac index (l/min/m2)	0.110*	0.146*	0.04	−0.164*	0.091	0.156*	−0.104*
PAWP (mmHg)	−0.009	−0.097*	0.067	0.088	0.019	−0.276**	0.151*
Pulmonary vascular resistance (wu)	−0.243**	−0.245**	−0.155*	0.220**	−0.240**	−0.155*	0.072
Transpulmonary pressure gradient (mmHg)	−0.069	−0.117*	−0.021	0.078	−0.170*	−0.011	0.027
Diastolic pulmonary gradient (mmHg)	0.055	−0.029	−0.033	−0.066	0.090	−0.103	0.017

RER, respiratory exchange ratio; PAWP, pulmonary arterial wedge pressure; VE/VCO2 - minute ventilation to carbon dioxide output.

**p < 0.001.

*p < 0.05.

**Figure 3:**
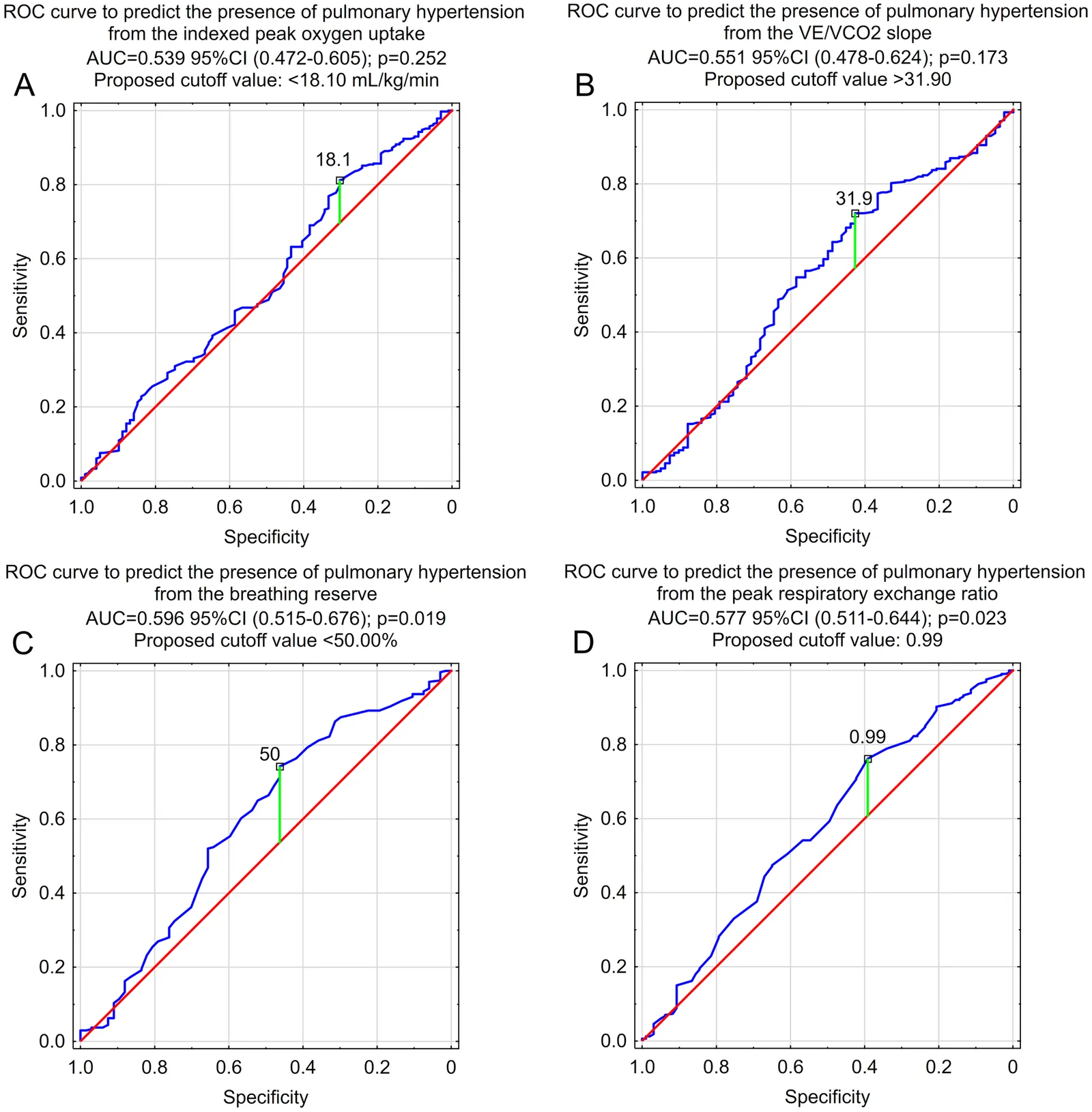
Receiver operating characteristic curves for prediction of pulmonary hypertension presence using indexed peak oxygen uptake **(A)**, VE/VCO2 slope **(B)**, breathing reserve at peak exercise **(C)** and peak respiratory exchange ratio **(D)**.

Multivariable analysis showed that indicators of functional capacity significantly improved the prediction of an unfavorable pulmonary hemodynamic, namely PH with PVR >3 WU phenotype, when added to the optimal model based on clinical and laboratory variables (for indexed oxygen uptake ΔAIC = 6.44; *p* < 0.005 and for VE/VCO2 slope ΔAIC = 7.47; *p* = 0.003) (Table 5). It revealed no additional prognostic value of indexed oxygen uptake or VE/VCO2 for presence of PH (Table 6). Breathing reserve did not improve prediction of PH presence or PH with PVR >3 WU. Receiver operating characteristic curve analysis showed no significant predictive value of indexed peak oxygen uptake or VE/VCO₂ slope for PH (Figure 3). In contrast, breathing reserve and peak respiratory exchange ratio demonstrated weak predictive ability for PH, with AUC values of 0.596 and 0.577, respectively.

**Table 5:** Prediction of PH with pulmonary vascular resistance >3 wood units using multivariable logistic regression model developed with best subset selection approach using akaike criterion (AIC) from following variables: age, LVEF, ln(BNP), GFR MDRD, sex, ischemic etiology, atrial fibrillation, diabetes mellitus, BMI, NYHA class III or IV. Then, model was augmented with selected CPET findings: indexed peak oxygen uptake, VE/VCO2 slope, breathing reserve (one at once).

Parameter	OR (95%CI) in multivariate model	Wald statistic	*p*-value	AIC	R2	AUC
Ln(BNP-pg/mL)	2.421 (1.723–3.403)	25.919	<0.001	324.79	0.232	0.756
Ischemic etiology	2.540 (1.432–4.502)	10.171	0.001			
Diabetes mellitus	2.023 (1.091–3.751)	5.003	0.025			
Atrial fibrillation	1.729 (0.970–3.048)	3.441	0.064			
GFR MDRD (mL/min/1.73m2)	0.989 (0.976–1.003)	2.432	0.119			
LVEF (%)	1.033 (0.990–1.078)	2.245	0.134			
Augmentation of the model with CPET variables (one at once)						
Parameter	OR (95%CI) in multivariate model	Wald statistic	*p*-value	AIC	R2	AUC
Indexed peak oxygen uptake (mL/kg/min)	0.896 (0.830–0.967)	7.907	0.005	318.35	0.265	0.776
VE/VCO2 slope	1.046 (1.015–1.078)	8.677	0.003	317.32	0.269	0.778
Breathing reserve (%)	0.991 (0.975–1.007)	1.316	0.251	325.47	0.237	0.757

OR, odds ratio; Ln(BNP-pg/mL), natural logarithm of B-type natriuretic peptide, LVEF, left ventricular ejection fraction; eGFR, glomerular filtration rate estimated by the Modification of Diet in Renal Disease (MDRD) equation; CAD, coronary artery disease; AF, atrial fibrillation (paroxysmal, permanent or persistent); DM, diabetes mellitus.

**Table 6:** Prediction of PH using multivariable logistic regression model developed with best subset selection approach using akaike criterion (AIC) from following variables: age, LVEF, ln(BNP), GFR MDRD, sex, ischemic etiology, atrial fibrillation, diabetes mellitus, body mass index, NYHA class III or IV. Then, model was augmented with selected CPET findings: indexed peak oxygen uptake, VE/VCO2 slope, breathing reserve (one at once).

Parameter	OR (95%CI) in multivariate model	Wald statistic	*p*-value	AIC	R2	AUC
Ln(BNP-pg/mL)	3.196 (2.287–4.466)	46.352	<0.001	240.80	0.357	0.824
Body mass index (kg/m2)	1.127 (1.032–1.230)	7.099	0.008			
GFR MDRD (mL/min/1.73m2)	0.967 (0.982–0.997)	5.479	0.019			
Age (years)	0.969 (0.932–1.008)	2.413	0.120			
Atrial fibrillation	1.713 (0.856–3.429)	2.321	0.128			
Augmentation of the model with CPET variables (one at once)						
Parameter	OR (95%CI) in multivariate model	Wald statistic	*p*-value	AIC	R2	AUC
Indexed peak oxygen uptake (mL/kg/min)	1.022 (0.940–1.112)	0.257	0.612	242.55	0.358	0.823
VE/VCO2 slope	0.986 (0.954–1.078)	0.706	0.401	242.11	0.360	0.824
Breathing reserve (%)	0.999 (0.980–1.019)	0.007	0.931	242.80	0.357	0.823

## 4 Discussion

To our knowledge, this study is the first to evaluate the association between RHC and cardiopulmonary exercise testing in such a large and homogeneous cohort of patients with heart failure. In addition, it provides insight into the clinical and hemodynamic characteristics of patients with pulmonary hypertension due to left heart disease.

No differences were observed between patients with and without PH for established CPET parameters: indexed peak oxygen uptake and VE/VCO₂ slope. PH could be expected to worsen exercise capacity, particularly given its association with lower LVEF and worse NYHA functional class in the study population. On the other hand, CPET parameters were correlated with several clinically relevant RHC measures, including cardiac index, PVR, and mean PAP, in line with previous studies (10–13). This finding may be explained by the fact that key CPET variables, through their reflection of abnormalities in ventilation–perfusion matching, can identify HF patients with elevated pulmonary pressure and pulmonary vascular resistance, but not necessarily those who meet the formal diagnostic criteria for PH, given the relatively low cutoff value of mean PAP >20 mmHg according to the European Society of Cardiology guidelines (3). More detailed analysis showed that functional capacity was significantly worse in patients with mean PAP >40 mmHg or in those with PH and PVR >3 WU. No significant differences in indexed peak oxygen uptake or VE/VCO₂ slope were observed until mean PAP or PVR reached those thresholds. In multivariable analysis, indexed peak oxygen uptake and VE/VCO₂ slope did not improve the prediction of PH presence. In contrast, indexed peak oxygen uptake and the VE/VCO₂ slope significantly improved the prediction of a more severe pulmonary vascular phenotype, defined as PH with PVR >3 WU. This threshold may be more clinically relevant, particularly in the context of heart transplantation candidacy (14). As a result, CPET parameters may help estimate selected hemodynamic abnormalities and potentially reduce the need for invasive testing and its associated risks. However, the observed predictive values of CPET parameters were moderate and clearly weaker than those of natriuretic peptides, indicating that CPET may play only a secondary role in estimating PH in the setting of HFrEF. Notably, the inclusion of patients with severely reduced LVEF evaluated for heart transplantation may also explain the lack of differences in functional capacity between patients with and without PH.

Two CPET parameters, lower breathing reserve and higher peak respiratory exchange ratio, demonstrated significant differences between HFrEF patients with and without PH. Although breathing reserve was correlated with most RHC parameters, the observed correlations were relatively weak. In multivariable analysis it did not improve prediction of either the presence of PH, PH with PVR >3 WU or PAP >40 mmHg. Its inclusion resulted in only a very modest improvement in the prediction of mean PAP≥25 mmHg (*Δ*AIC = 0.98; *p* = 0.091), Therefore, the study did not demonstrate usefulness of breathing reserve for predicting pulmonary circulation abnormalities in HFrEF patients. Generally, most correlations presented in the study are weak despite being statistically significant. Therefore, their results should be interpreted with caution.

The utility of noninvasive testing, including CPET, has been extensively investigated in the HF population and is now well established in the evaluation of clinical status and prognosis (1, 15, 16). The role of peak oxygen uptake in assessing disease severity and predicting adverse events is well recognized (16, 17). The VE/VCO₂ slope is a marker of excessive exercise ventilation and is also an important prognostic factor in patients with HF (18, 19). It may provide prognostic information beyond peak oxygen uptake because it is less dependent on patient effort, which is a known limitation of peak oxygen uptake (18, 19). Although the clinical value of these variables is well established, their role in identifying pulmonary hypertension remains limited.

Previous studies have demonstrated an association between VE/VCO₂ slope and ventilation–perfusion coupling in the lungs. Most prior research has focused on the use of CPET in precapillary PH, primarily pulmonary arterial hypertension (20–25). These studies showed good predictive value of VE/VCO₂ for differentiating patients with and without PH, as well as significant correlations between RHC and CPET findings (20–25). However, PH in patients with HFrEF represents a different clinical scenario, as individuals with HFrEF without PH may also exhibit markedly reduced exercise capacity and advanced HF (1). In contrast, in pulmonary arterial hypertension, elevated pulmonary vascular resistance is typically the primary determinant of reduced exercise tolerance.

Two studies have evaluated patients with PH due to left heart disease who underwent both CPET and invasive RHC, with study populations consisting predominantly of patients with HF and preserved ejection fraction. The study by Zhong et al. included 90 patients with PH due to left heart disease and examined the ability of CPET parameters to differentiate isolated postcapillary PH from combined pre- and postcapillary PH (10). The authors demonstrated good predictive value of VE/VCO₂ for identifying a precapillary component and reported correlations between RHC and CPET parameters, with the strongest association observed between VE/VCO₂ and PVR (10). However, only 4.4% of the study population had HFrEF, and the mean LVEF was 67.5%, in contrast to the homogeneous HFrEF cohort analyzed in this study. In addition, patients without PH were not included for comparison, and correlations were limited to a small number of RHC parameters, including PVR, diastolic pulmonary gradient, and transpulmonary pressure gradient (10). In another study, invasive RHC parameters were correlated with VE/VCO₂ slope in patients with HFpEF (11). That study included only 88 patients with LVEF>=45% compared with 428 patients with HFrEF. In this study, VE/VCOs was associated with mean PAP and PVR but not with the presence of PH, a finding consistent with the results of this study (11).

Additionally, two small studies correlated CPET parameters with echocardiographic estimates of right ventricular function and systolic PAP in HFrEF (12, 13). In the study by Methvin et al., systolic PAP estimated by echocardiography was correlated with higher VE/VCO₂ in patients with HFrEF (13). Similarly, in the study by Legris et al., elevated systolic PAP and, more specifically, a reduced tricuspid annular plane systolic excursion–to–systolic PAP ratio were associated with lower indexed peak oxygen uptake and higher VE/VCO₂ (12). These findings are consistent with the results of this study. However, in the above-mentioned studies (12, 13), systolic PAP was estimated using Doppler echocardiography based on the modified Bernoulli equation, with clinically assessed right atrial pressure added to the calculated gradient to derive systolic PAP. This approach is associated with a substantial risk of misestimating true pulmonary pressures (3, 26, 27). In contrast, in this study, all right heart pressures were measured directly by right heart catheterization, providing greater accuracy.

The presence of PH was associated with worse NYHA functional class, lower LVEF, higher concentrations of natriuretic peptides, and a higher prevalence of comorbidities, including diabetes mellitus and chronic kidney dysfunction, indicating more advanced disease and poorer clinical status in patients with PH. However, due to the observational nature of the study, causal relationships cannot be inferred.

## 5 Study limitations

It is a single-center study that enrolled a very homogeneous population of relatively young patients with HFrEF (median age, 55 years) with severely reduced LVEF, comprising mostly men. It should be noted that the study included mainly patients evaluated for heart transplantation or long-term mechanical circulatory support, which limits the generalizability to the broader HFrEF population or all patients with PH due to left heart disease. The sex composition of the study population is related to the inclusion of predominantly younger patients with HFrEF, as men represent the majority of patients with HFrEF aged 40–59 and 60–79 years (28). This is similar to many clinical trials (29, 30) and other studies in the field that enrolled HFrEF participants (12, 13). Importantly CPET and especially RHC are most commonly performed in younger patients with HFrEF, as they are essential for evaluation for heart transplantation and mechanical circulatory support. The study was conducted before the introduction of SGLT2 inhibitors into HF therapy; therefore, these agents were very rarely used (<5%) and were not analyzed. On the other hand, the study includes data collected over a long period, which was necessary to enroll a sufficient number of eligible patients. Data on end-tidal oxygen pressure (PETO₂) and end-tidal carbon dioxide pressure (PETCO₂) were not consistently available in the present cohort; therefore, these parameters could not be included in the analysis. Their potential relationship with invasive hemodynamic indices should be evaluated in future studies.

## 6 Conclusions

The incidence of PH increases as the clinical condition of patients with HF worsens. Noninvasive CPET parameters, including peak oxygen uptake, VE/VCO₂ slope, and breathing reserve, correlate with invasive RHC findings. Peak oxygen uptake and VE/VCO₂ slope did not improve prediction of PH, however they allowed more accurate prediction of more severe PH phenotype with PVR >3 WU. Therefore, noninvasive testing may help identify patients with HF who are at risk of hemodynamic abnormalities associated with PH, but it cannot replace invasive assessment by RHC. Patients with PH secondary to HFrEF have worse NYHA functional class, lower LVEF, higher concentrations of natriuretic peptides, and a higher prevalence of chronic kidney dysfunction and diabetes mellitus compared with patients without PH.

## Data Availability

The original contributions presented in the study are included in the article/Supplementary Material, further inquiries can be directed to the corresponding author.

## References

[1] McDonagh TA Metra M Adamo M Gardner RS Baumbach A Böhm M. 2021 ESC guidelines for the diagnosis and treatment of acute and chronic heart failure: developed by the task force for the diagnosis and treatment of acute and chronic heart failure of the European Society of Cardiology (ESC). with the special contribution of the heart failure association (HFA) of the ESC. Eur J Heart Fail. (2022) 24:4–131. 10.1002/ejhf.2333 35083827

[2] Hoeper MM Humbert M Souza R Idrees M Kawut SM Sliwa-Hahnle K. A global view of pulmonary hypertension. Lancet Respir Med. (2016) 4:306–22. 10.1016/S2213-2600(15)00543-3 26975810

[3] Humbert M Kovacs G Hoeper MM Badagliacca R Berger RMF Brida M. 2022 ESC/ERS guidelines for the diagnosis and treatment of pulmonary hypertension. Eur Heart J. (2022) 43:3618–731. 10.1093/eurheartj/ehac237 36017548

[4] Ghio S Gavazzi A Campana C Inserra C Klersy C Sebastiani R. Independent and additive prognostic value of right ventricular systolic function and pulmonary artery pressure in patients with chronic heart failure. J Am Coll Cardiol. (2001) 37:183–8. 10.1016/S0735-1097(00)01102-5 11153735

[5] Grigioni F Potena L Galiè N Fallani F Bigliardi M Coccolo F. Prognostic implications of serial assessments of pulmonary hypertension in severe chronic heart failure. J Heart Lung Transplant. (2006) 25:1241–6. 10.1016/j.healun.2006.06.015 17045937

[6] Peled Y Ducharme A Kittleson M Bansal N Stehlik J Amdani S. International society for heart and lung transplantation guidelines for the evaluation and care of cardiac transplant candidates-2024. J Heart Lung Transplant Off Publ Int Soc Heart Transplant. (2024) 43:1529–1628e54. 10.1016/j.healun.2024.05.010 39115488

[7] Betz J Jia K Lilly S Marmagkiolis K Mazzaferri EL Boudoulas KD. Bleeding complications related to right heart catheterization in the setting of elevated INR. J Invasive Cardiol. (2018) 30(5):191–4. PMID: 29440623

[8] Glaab T Taube C. Practical guide to cardiopulmonary exercise testing in adults. Respir Res. (2022) 23:9. 10.1186/s12931-021-01895-6 35022059 PMC8754079

[9] Ishihara S Hiramitsu S Kanaoka K Taki M Nakagawa H Ueda T. New conversion formula between B-type natriuretic peptide and N-terminal-pro-B-type natriuretic peptide- analysis from a multicenter study. Circ J. (2022) 86:2010–8. 10.1253/circj.CJ-22-0032 35613887

[10] Zhong X Tang J Jiang R Yuan P Zhao Q Gong S. The predictive value of minute ventilation versus carbon dioxide production in pulmonary hypertension associated with left heart disease. Ann Transl Med. (2021) 9:351. 10.21037/atm-21-366 33708978 PMC7944330

[11] Klaassen SHC Liu LCY Hummel YM Damman K Van Der Meer P Voors AA. Clinical and hemodynamic correlates and prognostic value of VE/VCO2 slope in patients with heart failure with preserved ejection fraction and pulmonary hypertension. J Card Fail. (2017) 23:777–82. 10.1016/j.cardfail.2017.07.397 28736291

[12] Legris V Thibault B Dupuis J White M Asgar AW Fortier A. Right ventricular function and its coupling to pulmonary circulation predicts exercise tolerance in systolic heart failure. ESC Heart Fail. (2022) 9:450–64. 10.1002/ehf2.13726 34953062 PMC8788036

[13] Methvin AB Owens AT Emmi AG Allen M Wiegers SE Dries DL. Ventilatory inefficiency reflects right ventricular dysfunction in systolic heart failure. Chest. (2011) 139:617–25. 10.1378/chest.10-0318 20688926

[14] Beyersdorf F Schlensak C Berchtold-Herz M Trummer G. Regression of “fixed” pulmonary vascular resistance in heart transplant candidates after unloading with ventricular assist devices. J Thorac Cardiovasc Surg. (2010) 140:747–9. 10.1016/j.jtcvs.2010.05.042 20850652

[15] Heidenreich PA Bozkurt B Aguilar D Allen LA Byun JJ Colvin MM. 2022 AHA/ACC/HFSA guideline for the management of heart failure: a report of the American College of Cardiology/American Heart Association joint committee on clinical practice guidelines. J Am Coll Cardiol. (2022) 79:e263–421. 10.1016/j.jacc.2021.12.012 35379503

[16] Corrà U Agostoni PG Anker SD Coats AJS Crespo Leiro MG De Boer RA. Role of cardiopulmonary exercise testing in clinical stratification in heart failure. A position paper from the committee on exercise physiology and training of the heart failure association of the European Society of Cardiology. Eur J Heart Fail. (2018) 20:3–15. 10.1002/ejhf.979 28925073

[17] Nadruz W West E Sengeløv M Santos M Groarke JD Forman DE. Prognostic value of cardiopulmonary exercise testing in heart failure with reduced, midrange, and preserved ejection fraction. J Am Heart Assoc. (2017) 6:e006000. 10.1161/JAHA.117.006000 29089342 PMC5721737

[18] Poggio R Arazi HC Giorgi M Miriuka SG. Prediction of severe cardiovascular events by VE/VCO2 slope versus peak VO2 in systolic heart failure: a meta-analysis of the published literature. Am Heart J. (2010) 160:1004–14. 10.1016/j.ahj.2010.08.037 21146651

[19] Sarullo FM Fazio G Brusca I Fasullo S Paterna S Licata P. Cardiopulmonary exercise testing in patients with chronic heart failure: prognostic comparison from peak VO2 and VE/VCO2 slope. Open Cardiovasc Med J. (2010) 4:127–34. 10.2174/1874192401004010127 20657715 PMC2908890

[20] Luo Q Yu X Zhao Z Zhao Q Ma X Jin Q. The value of cardiopulmonary exercise testing in the diagnosis of pulmonary hypertension. J Thorac Dis. (2021) 13:178–88. 10.21037/jtd-20-1061b 33569198 PMC7867820

[21] Correale M Tricarico L Ferraretti A Monaco I Concilio M Padovano G. Cardiopulmonary exercise test predicts right heart catheterization. Eur J Clin Invest. (2017) 47. 10.1111/eci.12851 29082512

[22] Correale M Monaco I Ferraretti A Tricarico L Sicuranza M Gallotta AM. Ventilatory power, a cardiopulmonary exercise testing parameter for the prediction of pulmonary hypertension at right heart catheterization. Int J Cardiol Heart Vasc. (2020) 28:100513. 10.1016/j.ijcha.2020.100513 32346602 PMC7178492

[23] Ptaszyńska-Kopczyńska K Krentowska A Sawicka E Skoneczny A Jasiewicz M Knapp M. The strengths and weaknesses of non-invasive parameters obtained by echocardiography and cardiopulmonary exercise testing in comparison with the hemodynamic assessment by the right heart catheterization in patients with pulmonary hypertension. Adv Med Sci. (2017) 62:39–44. 10.1016/j.advms.2016.06.001 28187374

[24] Pezzuto B Badagliacca R Muratori M Farina S Bussotti M Correale M. Role of cardiopulmonary exercise test in the prediction of hemodynamic impairment in patients with pulmonary arterial hypertension. Pulm Circ. (2022) 12:e12044. 10.1002/pul2.12044 35506106 PMC9052996

[25] Zhao QH Wang L Pudasaini B Jiang R Yuan P Gong SG. Cardiopulmonary exercise testing improves diagnostic specificity in patients with echocardiography-suspected pulmonary hypertension. Clin Cardiol. (2016) 40:95–101. 10.1002/clc.22635 28244596 PMC6490376

[26] D'Alto M Di Maio M Romeo E Argiento P Blasi E Di Vilio A. Echocardiographic probability of pulmonary hypertension: a validation study. Eur Respir J. (2022) 60(2):2102548. 10.1183/13993003.02548-2021 34996833

[27] Praz F Borger MA Lanz J Marin-Cuartas M Abreu A Adamo M. 2025 ESC/EACTS guidelines for the management of valvular heart disease: developed by the task force for the management of valvular heart disease of the European Society of Cardiology (ESC) and the European association for cardio-thoracic surgery (EACTS). Eur Heart J. (2025) 46:4635–736. 10.1093/eurheartj/ehaf194 40878295

[28] Virani SS Alonso A Aparicio HJ Benjamin EJ Bittencourt MS Callaway CW. Heart disease and stroke statistics-2021 update: a report from the American Heart Association. Circulation. (2021) 143:e254–743. 10.1161/CIR.0000000000000950 33501848 PMC13036842

[29] Morgan H Sinha A Mcentegart M Hardman SM Perera D. Evaluation of the causes of sex disparity in heart failure trials. Heart Br Card Soc. (2022) 108:1547–52. 10.1136/heartjnl-2021-320696 PMC9484380 35361671

[30] Reza N Gruen J Bozkurt B. Representation of women in heart failure clinical trials: barriers to enrollment and strategies to close the gap. Am Heart J Plus Cardiol Res Pract. (2022) 13:100093. 10.1016/j.ahjo.2022.100093 PMC8890694 35243454

