# Supplementary material for: Relationship between right heart catheterization and exercise testing in heart failure patients with and without pulmonary hypertension – cross-sectional study
Source: Front Cardiovasc Med. 2026 Aug 18;13:1812393. doi: 10.3389/fcvm.2026.1812393 (PMC13529533; doi:10.3389/fcvm.2026.1812393)
Supplement: Supplementary file 1 [file Table1.docx]

Supplementary Table 1. Comparison of patients with mean pulmonary artery pressure (PAP) ≥25 mmHg versus those with mean PAP <25 mmHg.

| Variable | Patients with mean PAP ≥25 mmHg (N=292) | Patients with mean PAP <25 mmHg (N=136) | p value |
| --- | --- | --- | --- |
| Age (years) | 55 (46-60) | 54 (44-59) | 0.284 |
| Female sex | 30 (10.3%) | 27 (19.8%) | 0.007 |
| Body mass index (kg/m2) | 27.18 (3.92) | 26.49 (4.23) | 0.096 |
| Ischemic etiology | 150 (51%) | 62 (45.6%) | 0.265 |
| LVEF (%) | 20 (15-25) | 25 (15-28) | 0.002 |
| NYHA class III/IV | 208 (71.2%) | 78 (57.4%) | 0.005 |
| BNP (pg/mL) | 587 (330-1055) | 201 (86-483) | <0.001 |
| NT-proBNP (pg/mL) | 2404 (1400-4652) | 1397 (483-2987) | <0.001 |
| GFR MDRD (ml/min/1.73m2) | 67.8 (20.3) | 75.9 (24.9) | <0.001 |
| Hemoglobin (mmol/L) | 8.94 (0.88) | 9.06 (0.92) | 0.216 |
| Peak oxygen uptake (ml/min) | 1172 (941-1390) | 1172 (892-1520) | 0.895 |
| Indexed peak oxygen uptake (ml/kg/min) | 14.5 (11.7-17.4) | 14.6 (12.0-18.7) | 0.220 |
| Tidal volume (ml) | 1587 (416) | 1520 (512) | 0.178 |
| VE/VCO2 slope | 37.3 (31.4-42.8) | 34.4 (28.0-41.4) | 0.015 |
| Oxygen uptake at anaerobic threshold (mL/min) | 838 (311) | 874 (290) | 0.420 |
| Breathing reserve (%) | 39 (30-50) | 46 (35-57) | <0.001 |
| Peak respiratory exchange ratio | 1.04 (0.99-1.09) | 1.02 (0.95-1.08) | 0.019 |

Categorical variables are presented as number of patients (% of each group), continuous variables with normal distribution are presented as mean (standard deviation), while other variables are depicted as median (lower quartile - upper quartile).

Abbreviations: BNP - B-type natriuretic peptide, eGFR - glomerular filtration rate, LVEF - left ventricular ejection fraction, MDRD - Modification of the Diet in Renal Disease, NYHA - New York Heart Association classification, NT-proBNP - N-terminal pro-B-type natriuretic peptide, PAP - pulmonary artery pressure, VE/VCO2 - ventilatory equivalent for carbon dioxide.

Supplementary Table 2. Comparison of patients with pulmonary vascular resistance (PVR) >3 Wood units and those with PVR ≤3 Wood units.

| Variable | Patients with PVR>3 Wood units (N=122) | Patients with PVR≤3 Wood units (N=306) | p value |
| --- | --- | --- | --- |
| Age (years) | 56 (49-61) | 54 (44-59) | 0.018 |
| Female sex | 19 (15.5%) | 38 (12.4%) | 0.386 |
| Body mass index (kg/m2) | 26.91 (3.90) | 26.98 (4.07) | 0.861 |
| Ischemic etiology | 73 (59.8%) | 139 (45.4%) | 0.007 |
| LVEF (%) | 20 (15-25) | 20 (15-25) | 0.219 |
| NYHA class III/IV | 93 (76.2%) | 193 (63.1%) | 0.009 |
| BNP (pg/mL) | 679 (418-1134) | 360 (169-760) | <0.001 |
| NT-proBNP (pg/mL) | 2419 (1510-4334) | 1876 (936-3941) | 0.058 |
| GFR MDRD (ml/min/1.73m2) | 64.0 (17.8) | 73.0 (23.3) | <0.001 |
| Hemoglobin (mmol/L) | 8.90 (0.95) | 9.01 (0.87) | 0.303 |
| Peak oxygen uptake (ml/min) | 1032 (841-1252) | 1248 (962-1544) | <0.001 |
| Indexed peak oxygen uptake (ml/kg/min) | 13.1 (10.8-15.3) | 15.2 (12.3-18.5) | <0.001 |
| Tidal volume (ml) | 1436 (394) | 1566 (464) | 0.002 |
| VE/VCO2 slope | 39.4 (33.4-45.9) | 34.9 (28.5-41.0) | <0.001 |
| Oxygen uptake at anaerobic threshold (mL/min) | 742 (281) | 894 (305) | 0.001 |
| Breathing reserve (%) | 38 (29-48) | 43 (32-53) | 0.036 |
| Peak respiratory exchange ratio | 1.04 (0.99-1.09) | 1.03 (0.97-1.09) | 0.198 |

Categorical variables are presented as number of patients (% of each group), continuous variables with normal distribution are presented as mean (standard deviation), while other variables are depicted as median (lower quartile - upper quartile).

Abbreviations: BNP - B-type natriuretic peptide, eGFR - glomerular filtration rate, LVEF - left ventricular ejection fraction, MDRD - Modification of the Diet in Renal Disease, NYHA - New York Heart Association classification, NT-proBNP - N-terminal pro-B-type natriuretic peptide, PVR - pulmonary vascular resistance, VE/VCO2 - ventilatory equivalent for carbon dioxide.

Supplementary Table 3. Characteristics of the study group stratified by pulmonary artery pressure. Continuous variables were compared with ANOVA or Kruskal-Wallis test according to the presence of normal distribution. Detailed results of post-hoc Tukey test for CPET results are presented below table.

| Variable | Patients with mean PAP ≤ 20 mmHg (N=99) (A) | Patients with mean PAP 21-30 mmHg (N=110) (B) | Patients with mean PAP 31-40 mmHg (N=118) (C) | Patients with mean PAP > 40 mmHg (N=101) (D) | p value |
| --- | --- | --- | --- | --- | --- |
| Age (years) | 54 (44-60) | 54 (43-59) | 55 (45-60) | 56 (49-61) | 0.489 |
| Female sex | 20 (20.2%) | 17 (15.4.2%) | 13 (11.0%) | 7 (6.9%) | 0.035 |
| Body mass index (kg/m2) | 26.43 (4.26) | 26.82 (4.00) | 26.80 (3.79) | 27.83 (4.02) | 0.081 |
| Ischemic etiology | 44 (44.4%) | 53 (48.2%) | 52 (44.1%) | 63 (62.4%) | 0.027 |
| LVEF (%) | 25 (20-27) | 25 (15-25) | 25 (15-25) | 25 (15-25) | 0.008 |
| NYHA III/IV | 53 (53.5%) | 74 (67.3%) | 82 (69.5%) | 77 (76.2%) | 0.006 |
| BNP (pg/mL) | 150 (83-368) | 372 (203-683) | 628 (345-1135) | 678 (435-1138) | <0.001 |
| NT-proBNP (pg/mL) | 921 (412-2100) | 1816 (992-3328) | 3241 (1632-5493) | 2419 (1820-5836) | <0.001 |
| GFR MDRD (ml/min/1.73m2) | 76.1 (25.4) | 73.1 (23.7) | 67.3 (19.5) | 64.8 (18.5) | <0.001 |
| Hemoglobin (mmol/L) | 9.05 (0.90) | 9.01 (0.87) | 8.94 (0.85) | 8.93 (0.96) | 0.742 |
| Peak oxygen uptake (ml/min) | 1179 (890-1524) | 1238 (962-1526) | 1198 (953-1391) | 1105 (896-1350) | 0.623 |
| Indexed peak oxygen uptake (ml/kg/min) | 15.58 (4.99) | 15.51 (4.79) | 15.10 (4.36) | 13.74 (3.98) | 0.013* |
| Tidal volume (ml) | 1548.1 (501.7) | 1549 (438) | 1607 (417) | 1572 (419) | 0.626 |
| VE/VCO2 slope | 34.1 (28.1-42.5) | 34.8 (29.1-40.8) | 36.8 (30.7-43.0) | 38.5 (33-43.1) | 0.032** |
| Oxygen uptake at anaerobic threshold (mL/min) | 881.0 (306.1) | 872 (325) | 848 (294) | 794 (301) | 0.478 |
| Breathing reserve (%) | 44.6 (17.4) | 42.7 (16.7) | 38.7 (16.9) | 36.9 (16.9) | 0.004* |
| Peak respiratory exchange ratio | 1.02 (0.95-1.07) | 1.02 (0.97-1.09) | 1.05 (0.99-1.10) | 1.04 (0.99-1.09) | 0.085 |

Categorical variables are presented as number of patients (% of each group), continuous variables with normal distribution are presented as mean (standard deviation), while other variables are depicted as median (lower quartile - upper quartile).

*Tukey post-hoc tests results: - Indexed peak oxygen uptake (ml/kg/min): A vs. B p=0.999, A vs. C p=0.864, B vs. C p=0.906, A vs. D p=0.022; B vs. D p=0.025; C vs. D p=0.121, - Breathing reserve (%): A vs. B p=0.902, A vs. C p=0.115, B vs. C p=0.349, A vs. D p=0.029; B vs. D p=0.111; C vs. D p=0.896,

** Dunn post-hoc tests results VE/VCO2 slope: A vs. B p=1.000, A vs. C p=1.000, B vs. C p=1.000, A vs. D p=0.086; B vs. D p=0.055; C vs. D p=1.000,

Abbreviations: BNP - B-type natriuretic peptide, eGFR - glomerular filtration rate, LVEF - left ventricular ejection fraction, MDRD - Modification of the Diet in Renal Disease, NYHA - New York Heart Association classification, NT-proBNP - N-terminal pro-B-type natriuretic peptide, PAP – pulmonary artery pressure, VE/VCO2 - ventilatory equivalent for carbon dioxide.
